# Uncovering the role of microRNAs in esophageal cancer: from pathogenesis to clinical applications

**DOI:** 10.3389/fphar.2025.1532558

**Published:** 2025-01-29

**Authors:** Zhenglin He, Yishuo Ji, Yutong Yuan, Tianfang Liang, Chenglin Liu, Yiping Jiao, Yimeng Chen, Yiming Yang, Liang Han, Yue Hu, Xianling Cong

**Affiliations:** ^1^ China-Japan Union Hospital of Jilin University, Jilin University, Changchun, China; ^2^ The First Bethune Hospital of Jilin University, Jilin University, Changchun, China; ^3^ The Second Bethune Hospital of Jilin University, Jilin University, Changchun, China; ^4^ Department of Cell Biology, College of Basic Medical Science, Jilin University, Changchun, China; ^5^ Department of Pathology, China-Japan Union Hospital of Jilin University, Changchun, China; ^6^ Department of Biobank, China-Japan Union Hospital of Jilin University, Changchun, China; ^7^ Department of Dermatology, China-Japan Union Hospital of Jilin University, Changchun, China

**Keywords:** microRNAs, esophageal cancer, pathogenesis, diagnosis, prognosis, treatment resistance

## Abstract

Esophageal cancer (EC) presents substantial therapeutic challenges due to its high mortality rate and the tendency for diagnosis at advanced stages. Acknowledging the constraints of the existing current treatment paradigm, there is a pressing need for the development of innovative, targeted therapeutic strategies to surpass the current stagnation in survival rate improvements. Recently, microRNAs (miRNAs) have attracted significant attention for their capacity to regulate gene expression at the post-transcriptional level, thereby influencing various cellular processes. In this review, we provide a comprehensive analysis of the role of miRNAs in EC, emphasizing their mechanisms in tumorigenesis, their potential as diagnostic and prognostic biomarkers, and their influence on resistance to therapy. We elucidate how miRNAs modulate oncogenic pathways and tumor suppressor genes, influencing EC cell behavior and treatment outcomes. By integrating insights from genetic sequencing and molecular biology, we identify key miRNAs that promote or inhibit EC progression and treatment resistance. This review highlights critical need for an enhanced understanding of miRNAs in EC, advocating for their integration into therapeutic strategies.

## 1 Introduction

Esophageal cancer (EC) is a malignancy that originates from the epithelial cells of the esophagus. As reported in the 2022 World Cancer Report (GLOBOCAN), the incidence of EC has increased to 510,716 new cases, representing 2.6% of all newly diagnosed cancer cases, with 445,129 deaths attributed to the disease, making it the seventh leading cause of cancer-related mortality (Bray et al., 2024). Despite advancements in diagnostic and therapeutic strategies, the prognosis for EC remains poor, primarily due to its late-stage diagnosis and high metastatic potential (Pennathur et al., 2013). The current standard treatment options, which include surgical or endoscopic resection and chemoradiation, are insufficient, highlighting the need for further investigation into the molecular mechanisms of EC to identify novel biomarkers and develop targeted therapies (Rustgi and El-Serag, 2014; Ohashi et al., 2015; Yang et al., 2020).

MicroRNAs (miRNAs) are small non-coding RNAs that play a crucial role in the regulation of gene expression, impacting cellular processes such as proliferation, differentiation, metabolism, and apoptosis (Chen et al., 2022; Alexander et al., 2010; Berindan-Neagoe et al., 2014). They are pivotal in the process of carcinogenesis by modulating the genes expression. Dysregulation of miRNAs has been observed in various cancers (Vannini et al., 2018; Chakrabortty et al., 2023; Cho et al., 2020). Specific miRNAs, such as miR-21, have been implicated in the growth and progression of multiple malignancies (Gan et al., 2024; Jiang et al., 2023; Hui et al., 2015). Certain miRNAs, such as miR-25, miR-99a, miR-133a, and miR-133b, exhibit potential as diagnostic biomarkers for EC, while others including miR-21, miR-27b, miR-126, miR-143, and miR-145, are promising candidates for both diagnostic and prognostic marker applications (Wang et al., 2018; Sakai et al., 2013). The distinct expression patterns of miRNAs in EC highlight their involvement in the pathogenesis of the disease (Hemmatzadeh et al., 2016; Cui and Cheung, 2021). Nevertheless, a comprehensive understanding of their novel roles in EC is still developing.

Gaining insights into the functions of miRNAs in cancer biology holds significant promise for the development of innovative therapeutic strategies. This review delves into the critical roles of miRNAs in EC, detailing their impact on the disease pathogenesis, their potential as prognostic and diagnostic biomarkers, and their involvement in mechanisms of treatment resistance. It synthesizes the current understanding and projects prospective research directions, emphasizing the need for further investigation into the therapeutic potential of miRNAs and their integration into clinical practice.

## 2 Epigenetic alterations and esophageal cancer

Within the field of oncology, the enduring adverse effects associated with conventional cancer therapies pose a substantial challenge. Consequently, there is a growing interest in developing new therapeutic strategies that can target multiple pathways, demonstrate high efficacy, and have minimal side effects and costs. In this regard, epigenetic modification emerges as a promising approach for EC treatment, with pivotal mechanisms such as DNA methylation, histone modifications, and ncRNA regulation presenting novel therapeutic opportunities (Liu et al., 2023).

Non-coding RNAs (NcRNAs), once considered “transcriptional noise,” are now recognized for their crucial roles in cancer biology (Costa, 2005; Anastasiadou et al., 2018). NcRNAs include miRNAs, long non-coding RNAs (lncRNAs), and circular RNAs (circRNAs), which play integral roles in cancer progression through their complex regulatory networks. NcRNAs participate in intricate cross-regulatory networks, wherein miRNAs target lncRNAs to modulate their expression. Concurrently, lncRNAs and circRNAs function as miRNA sponges, thereby influencing gene expression by competing for miRNA binding sites (Xiao et al., 2019). The interplay is essential for EC processes such as proliferation, apoptosis, metastasis, and chemoradiotherapy response (Feng et al., 2019). For instance, XIST promotes cell proliferation and migration by sponging miR-129-5p and upregulating CCND1 (Wang H. et al., 2021). CircLPAR3 overexpression enhances tumor cell proliferation, migration, and invasion by sponging miR-375 and miR-433, which results in the upregulation of HMGB1 (Cheng et al., 2020). Furthermore, LncRNAs and circRNAs also collaborate with miRNAs to regulate genes expression. For instance, LncRNA SNHG6 downregulates the miR-101-3p/EZH2 pathway in esophageal squamous cell carcinoma (ESCC) (Zhang et al., 2022a), and circNTRK2 promotes cell proliferation, migration, and EMT by sponging miR-140-3p and upregulating NRIP1 (Chen X. et al., 2020). Additionally, lncRNAs can impact epigenetic modifications by interacting with miRNAs, as shown by lncRNA brain-derived neurotrophic factor antisense (BDNF-AS) targeting miR-214 and affecting DNA methylation and histone modification (Zhao et al., 2018). These interactions underscore the coordinated roles of lncRNAs, circRNAs, and miRNAs in EC progression.

**FIGURE 1:**
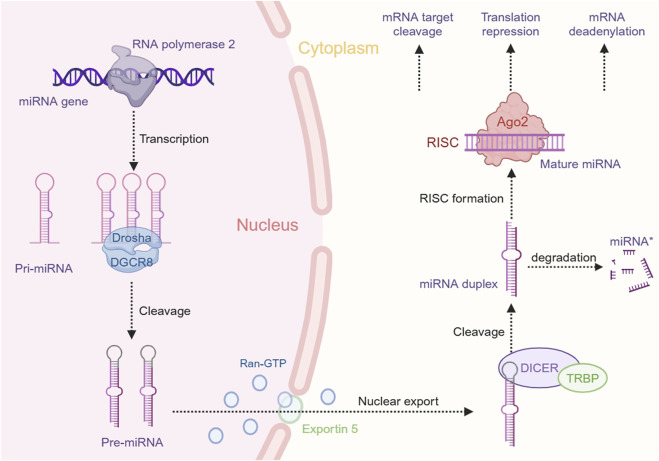
miRNA biogenesis and function in gene expression regulation. This schematic represents the intricate process of miRNA biogenesis and its role in the post-transcriptional regulation of gene expression within the cellular environment. The figure was created with BioRender.com.

**FIGURE 2:**
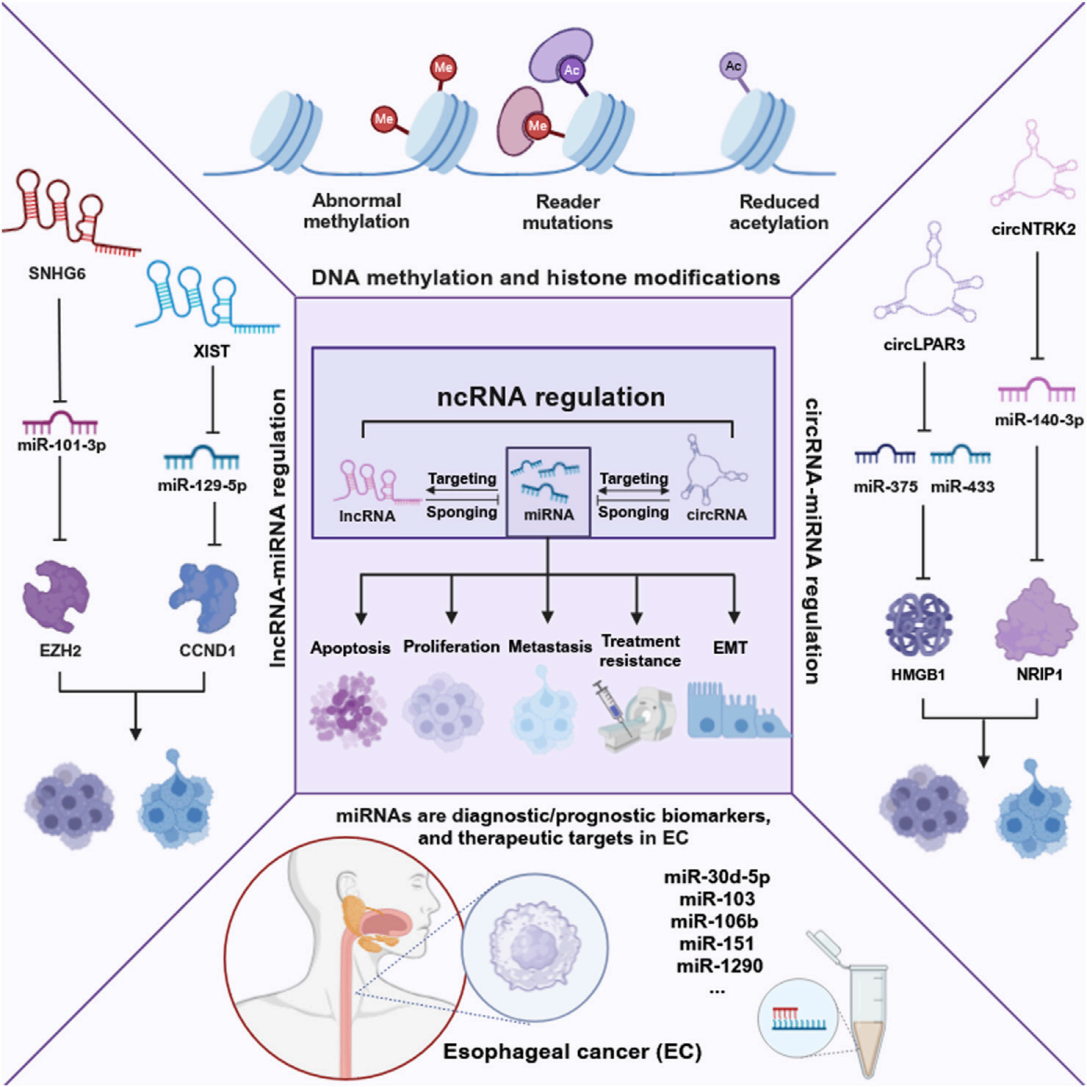
Epigenetic landscape in EC. This figure delineates the intricate network of epigenetic alterations and non-coding RNAs in EC, highlighting their roles in cancer biology. It illustrates the impact of DNA methylation, histone modifications, and especially the regulation by non-coding RNAs on cancer-related processes such as proliferation, apoptosis, metastasis, treatment resistance, and epithelial-mesenchymal transition (EMT). The figure was created with BioRender.com.

Among the various small ncRNAs mentioned, miRNAs are the most extensively studied in EC. MiRNAs, a class of small ncRNAs approximately 22 nucleotides in length, play a pivotal role in cancer biology (Zhang et al., 2014). Initially identified in *Caenorhabditis elegans*, miRNAs are transcribed by RNA polymerase II into primary miRNAs (pri-miRNAs), which are subsequently processed by the microprocessor complex, comprising Drosha and DGCR8, into precursor miRNAs (pre-miRNAs). These pre-miRNAs are exported to the cytoplasm, where they are cleaved by Dicer to produce mature miRNAs that incorporate into the RNA-induced silencing complex (RISC). The “seed” region of the miRNA, a 6- to 8-nucleotide segment, facilitates binding to mRNA targets, leading to their degradation or translational repression (Lee et al., 1993; Bartel, 2004; Shang et al., 2023) (Figure 1). Dysregulated miRNA expression patterns in EC, identified through microarray analysis, highlight their potential as oncogenes or tumor suppressors and their influence on EC-related processes. Liu et al. identified a total of 112 miRNAs in EC using the Cancer Genome Atlas (TCGA) database, with 38 being upregulated and 74 downregulated (Liu et al., 2020a). Some dysregulated miRNAs have been confirmed to influence the progression and development of EC, potentially serving as therapeutic targets (Zarrilli et al., 2021). For example, miR-148a-3p was found to suppress the expression of DNMT1, consequently inhibiting the proliferation and invasion of EC cells (Wang Y. et al., 2019). Conversely, overexpression of miR-191 promotes tumor proliferation and invasion by targeting the tumor suppressor EGR1 in ESCC (Gao et al., 2017). Wu et al. found that miR-375 targets XPR1, inhibiting cell proliferation, invasion, and migration in ESCC (Wu et al., 2021). Additionally, growing evidence suggests that circulating miRNAs in plasma and serum serve as potential biomarkers for noninvasive cancer diagnosis and prognosis. An 8-miRNA signature (including miR-103, miR-106b, miR-151) has been developed for non-invasive early detection of ESCC, with higher diagnostic accuracy than traditional biomarkers like SCC-Ag (Miyoshi et al., 2022). Furthermore, miR-30d-5p and miR-1290 have been identified as novel diagnostic and prognostic biomarkers for EC (Zhao Q. et al., 2022; Sun H. et al., 2019). These findings highlight the significant role of ncRNAs, especially miRNAs, in EC progression and their potential as therapeutic targets for future strategies (Figure 2).

## 3 MicroRNAs in pathogenesis of esophageal cancer

Advances in genetic sequencing and molecular biology have catalyzed significant progress in EC research. An expanding body of literature has identified numerous miRNAs with aberrant expression patterns that influence cellular processes by degrading target genes or inhibiting their expression. This modulation impedes EC cell proliferation, apoptosis, migration, and invasion, thereby affecting tumorigenesis and cellular mechanisms in EC (Xiao et al., 2024; Xue et al., 2022). The underlying mechanism of miRNAs exert a multifaceted impact by targeting various pathways involved in neoplastic processes, with significant implications for clinical diagnosis and treatment strategies. Table 1 encapsulates the aberrant expression of miRNAs in EC, their molecular targets, and biological functions, offering a comprehensive catalogue of potential biomarkers and therapeutic targets.

**TABLE 1:** miRNA profiles in the pathogenesis of EC.

Function mechanism		miRNAs	Expression	Target genes/pathways	Ref.
Cell Proliferation		miR-574-5p	↑	Inhibiting the expression of CTDSP1	Zhao et al. (2022b)
		miR-193a-3p	↑	Reducing the expression of ALKBH5 mRNA	Xue et al. (2021)
		miR-137	↓	Inhibiting the expression of EZH2 and PXN	Sun et al. (2019b)
		miR-150	↓	Inhibiting the expression of Gli1 and CyclinD1	Sun et al. (2017)
		miR-203	↓	Inhibiting the expression of CDK6 and E2F1	Zhang et al. (2015)
Cell apoptosis		miR-1290	↑	Inhibiting the expression of Bcl-2	Fang et al. (2022)
		miR-106b-5p miR-182 miR-183-5p	↑	Inhibiting the expression of HPGD	Shu et al. (2024), Yang et al. (2022)
		miR-204-5p	↓	Inhibiting the expression of Nestin mRNA	Luo et al. (2022a)
Tumor angiogenesis		miR-493-5p	↓	Inhibiting the expression of SP1 and SP3	Sharma et al. (2023)
		miR-18b-5p	↓	Inhibiting the expression of HIF-1α	Si et al. (2023)
Tumor metastasis	Cell adhesion movement	miR-375	↓	Inhibiting the expression of PRDX1	Wu et al. (2022b)
		miR-25	↑	Inhibiting the expression of E-cadherin	Liu et al. (2019)
		miR-595	↓	Inhibiting the expression of SEMA4D	Zhou et al. (2021a)
		miR-1294	↓	Inhibiting the expression of PBX3	Zhou et al. (2023)
		miR-200c	↑	Promoting the expression of EZH2	Nourmohammadi et al. (2022)
	Extracellular matrix degradation	miR-34a	↓	Inhibiting the expression of FOXM1	Nie et al. (2015)
		miR-181a-5p	↓	Diminishing the phosphorylation of ERK1/2	Wang et al. (2022a)
		miR-34c-3p	↓	Inhibiting the expression of FOS	Zhong et al. (2019)

### 3.1 Cell proliferation

Irregular progression of the cell cycle is a fundamental driver of unchecked cell proliferation, potentially leading to the malignant transformation characteristic of cancer. Specifically, miRNAs are involved in the regulation of mRNA encoding cyclin in ESCC cells, thereby influencing cell cycle progression. miR-137 is downregulated in ESCC, with CTCF binding to its promoter region to inhibit transcription (Xu et al., 2021). Furthermore, CTCF recruits PRC2 complexes to methylate the miR-137 promoter, exacerbating its suppression (Xu et al., 2014). Intriguingly, the overexpression of miR-137 has been shown to counteract the EMT. This effect is mediated through the targeting of enhancer of EZH2 and PXN (Sun J. et al., 2019; Jiang et al., 2018), thereby significantly modulating the behavior of tumor cells (Xu et al., 2014), resulting in inducing cell cycle arrest at G2/M. MiRNAs that can also cause cell cycle arrest in phase G2/M include miR-210, miR-142, miR-219-5p, miR-382, and so on (Li et al., 2014; Xiao et al., 2021; Ma, 2019; Feng et al., 2017). MiR-29b, miR-940, miR-150, miR-139-5p can induce cell cycle arrest in the G0/G1 phase (Guo et al., 2021; Wang H. et al., 2020; Sun et al., 2017; Liu et al., 2013). Among them, miR-150 can induce G0/G1 cell cycle arrest and weaken the proliferation of EC cells by targeting the downstream expression of Gli1 and Cyclin D1. In addition, Zhang et al. found that miR-203 negatively inhibits the expression of CDK6, and subsequently may reduce the expression of E2F1 through Rb phosphorylation, thereby causing the EC cell cycle to stagnate in the G1/S phase (Zhang et al., 2015). Zhao et al. confirmed that miR-574-5p promotes ESCC cell proliferation by binding to CTDSP1 mRNA’s 3'-UTR, miR-574-5p suppresses its expression, leading to decreased CTDSP1 protein levels and increased AKT phosphorylation, which is a hallmark of tumor cell survival and proliferation (Zhao C. et al., 2022). miR-193a-3p, targets ALKBH5 mRNA, reducing its expression which impacts the maturation of pri-miR-193a-3p by removing m6A modification, thus creating a positive feedback loop that enhances ESCC cell proliferation (Xue et al., 2021). As research into miRNAs advances, it is anticipated that additional miRNAs affecting the proliferation and cell cycle of EC will be identified, thereby providing a robust foundation for targeted therapies and early diagnostic strategies.

### 3.2 Cell apoptosis

Apoptosis, also known as programmed cell death, is a meticulously regulated process of cellular self-elimination triggered by alterations in internal and external conditions or by genetically encoded signals. Tumors represent a heterogeneous group of disorders characterized not only by abnormal cell growth and differentiation but also by disruptions in the regular process of cell apoptosis. Recent studies have demonstrated that miRNAs play a pivotal role in regulating tumor cell apoptosis. For instance, miR-1290 is notably upregulated in ESCC tissues compared to normal tissues (Fang et al., 2022). It plays a pivotal role in tumor progression by binding to circCDR1 (CDR1), which is typically involved in promoting apoptosis (Zhao et al., 2020). However, miR-1290 counteracts the tumor-suppressive effects of CDR1 by modulating the expression of apoptosis-related factors, including Bcl-2, Bax, and caspase-3, and facilitating tumor spread (Kim et al., 2014). Similar to miR-182/183-5p, miR-106b-5p directly binds to the 3'-UTR of HPGD to inhibit its expression (Shu et al., 2024; Yang et al., 2022). This microRNA also promotes cell cycle progression and proliferation by decreasing the proportion of cells in the G1 phase and increasing those in the S phase. It will further reduce esophageal adenocarcinoma (EAC) cell apoptosis by modulating the expression of Bax and Bcl-2 (Kan et al., 2009; Wu P. et al., 2022). On the contrary, Wang et al. demonstrated that miR-378a-3p can induce apoptosis in EAC cells by targeting UHRF1 (Wang et al., 2024). Nestin, an intermediate filament protein typically expressed in stem cells and some malignant tumor cells, is associated with the malignant characteristics of ESCC when highly expressed (Zhong et al., 2014). In addition, miR-204-5p has been found to directly target the 3'-UTR of Nestin mRNA, thereby continuously translating Nestin and inhibiting its expression to suppress tumor cell proliferation and promote apoptosis (Luo H. et al., 2022). The mechanisms through which different miRNAs influence EC apoptosis are diverse, however, the number of miRNAs identified to affect EC apoptosis remains limited. Further research is essential to elucidate the regulatory mechanisms of miRNAs on EC apoptosis.

### 3.3 Tumor angiogenesis

Tumor angiogenesis is a critical process in the proliferation and dissemination of cancer cells. The development of anti-angiogenic therapies focuses on disrupting the regulatory elements that govern the formation of blood vessels within tumors and their interactions. VEGF, αFGF, ANG and BDNF are key players in this process, acting as essential drivers of vascular growth. Thus, miRNAs can modulate tumor angiogenesis by directly targeting angiogenic factors, potentially offering a novel therapeutic target for cancer treatment (Choi et al., 2011; Zhang Y. et al., 2024; Li et al., 2022). For instance, Sharma et al. has found miR-493-5p in plasma exosomes of ESCC patients, where its expression is significantly reduced and negatively correlates with tumor T staging, LNM status, and tumor staging (Sharma et al., 2023). Moreover, miR-493-5p is capable of being transported from ESCC cells to human umbilical vein endothelial cells (HUVECs) through exosomes. This process effectively inhibits HUVEC proliferation, migration, and tubular structure formation, consequently diminishing angiogenesis. Furthermore, miR-493-5p directly targets and suppresses the expression of the transcription factors SP1 and SP3, which play crucial roles in tumor angiogenesis and proliferation (Situ et al., 2022). MiR-18b-5p interacts with hypoxia-inducible factor-1α (HIF-1α), which plays a role in malignant tumor development by mediating angiogenesis, cell metabolism, metastasis, and apoptosis (Luo P. et al., 2022; Zhang L. et al., 2024). Si et al. have identified that miR-18b-5p exerts its inhibitory effect on HIF-1α expression by directly targeting the 3'-UTR of the HIF-1α mRNA. When miR-18b-5p is inhibited by circ_ZNF778_006, a specific circRNA, the expression level of HIF-1α increases, thereby promoting tumor angiogenesis (Si et al., 2023). Understanding these regulatory mechanisms is pivotal for paving the way for advancing vascular-targeted EC therapy. Therapeutic strategies targeting myeloid cell-mediated tumor angiogenesis have the potential to alter the tumor microenvironment, thereby overcoming resistance to radiotherapy, chemotherapy, and immunotherapy. These insights are vital for developing more effective treatments for EC.

### 3.4 Tumor metastasis

EC exhibits a profound propensity for invasion, with its invasive and migratory characteristics serving as predictors of the tumor’s metastatic potential. Empirical investigations have demonstrated a substantial correlation between the specific expression patterns of miRNAs within EC tissues and the tumor’s capacity to invade and metastasize, thereby regulating the aggressive behavior of EC cells in a nuanced manner (Tian et al., 2024).

#### 3.4.1 Cell adhesion movement

The primary neoplastic cells undergo EMT, endowing them with the attributes necessary for metastasis. This transition is characterized by a reduction in the intercellular adhesion mediated by E-cadherin, alongside an upregulation of mesenchymal markers, such as vimentin and N-cadherin, ultimately leading to reduced cellular adhesion. These molecular alterations facilitate the pervasion of tumor cells into the adjacent tissues and their dissemination to distant organs (Sun et al., 2024). Wu et al. discovered miR-375 directly targets the 3'-UTR of the mRNA of PRDX1, inhibiting its expression (Wu K. et al., 2022). PRDX1 facilitates the depolymerization of primary cilia in ESCC cells by activating the HEF1-Aurora A-HDAC6 axis to foster the dissemination of the malignancy (Chen Q. et al., 2020). The expression of PRDX1 in ESCC cells is negatively correlated with the expression of miR-375. Overexpression of miR-375 significantly reduces the invasive potential of ESCC cells and decreases the expression of genes related to tumor cell migration and invasion, such as E-cadherin and N-cadherin (Wu K. et al., 2022). Similar to miR-375, a certain group of miRNAs such as miR-25, miR-595, miR-1294, and so on, could influence tumor spread through modulating E-cadherin (Liu et al., 2019; Zhou S. et al., 2021; Zhou et al., 2023). MiR-200c is able to affect the process of EMT by regulating EZH2, which affects the spread of tumor cells by regulating the expression of EMT related genes. In EZH2 silenced cells, the expression of mesenchymal markers such as vimentin, fibronectin, N-cadherin, and Zeb2 decreased, while the expression of E-cadherin and occludin increased. On the contrary, decreased expression of epithelial markers and increased expression of mesenchymal markers were observed in EZH2 overexpressing cells, thereby promoting the spread of tumor cells (Nourmohammadi et al., 2022).

#### 3.4.2 Extracellular matrix degradation

Proteolytic enzymes of the matrix metalloproteinase family are essential in the remodeling of the extracellular matrix, with their degradation being a prerequisite for the invasion and metastasis of malignant tumors. Researchers have elucidated mechanisms by which several miRNAs attenuate the invasive capacity of ESCC cells by modulating matrix metalloproteinases (MMPs). Yang et al. found increasing miR-34a could suppress FOXM1, a downstream target, which in turn decreased MMP-2 and MMP-9 levels, thereby inhibiting EC cell migration and invasion (Yang L. et al., 2017). Additionally, miR-181a-5p has been shown to reduce the phosphorylation of ERK1/2 via MEK1 downregulation, consequently inhibiting the expression of MMP-2 and MMP-9 (Wang M. et al., 2022). In parallel, miR-34a exerts its inhibitory effect on these matrix metalloproteinases by enhancing YY1 expression (Nie et al., 2015). A novel regulatory axis in which miR-34c-3p modulates the expression of MMP-1 and MMP-9 by directly targeting ZEP1 (Zhong et al., 2019). These studies revealed that miRNAs significantly influence the EMT process and the invasive behavior of EC cells. This insight into miRNA’s role is crucial for elucidating the disease’s progression and may pave the way for novel clinical strategies to combat EC’s recurrence and metastasis.

## 4 MicroRNAs in clinical applications of esophageal cancer

### 4.1 Diagnostic biomarkers in esophageal cancer

Early detection of EC significantly improves patient survival rates. However, EC frequently presents with non-specific clinical symptoms, and the current diagnostic gold standard—esophageal endoscopy with biopsy—is invasive and often results in diagnosis at advanced stages, thereby severely limiting treatment options and adversely affecting quality of life. The advent of miRNA research has identified certain miRNAs as specific serum biomarkers for EC diagnosis, offering a less invasive approach to early detection (Wu et al., 2024). For instance, MiR-493-5p was significantly elevated in ESCC patients, indicating that it could serve as a highly sensitive and specific molecular marker for ESCC (Xiao et al., 2024). In addition, high expression of miR-196a-5p in ESCC was closely related to its malignant phenotype and poor prognosis, serving it a potential diagnostic biomarker for ESCC (Xian et al., 2024). Kim et al. meticulously isolated exosomes from both normal esophageal epithelial cells and ESCC cell lines, identifying miR-205-5p and miR-429 as upregulated, and miR-375-3p and miR-483-3p as downregulated in ESCC. Subsequent validation in plasma samples from healthy control (HC) group and ESCC patients revealed that miR-205-5p and miR-429 were significantly increased, while miR-375-3p was decreased in patients, affording a diagnostic sensitivity and specificity of 72.5% and 70.0%, 60.0% and 60.0%, and 65.0% and 65.0%, respectively (Kim et al., 2022). Huang et al. detected the expression level of miR-4488, which was correlated with clinical pathological parameters such as TNM staging and lymph node metastasis in ESCC patients. It was highly expressed in the serum of ESCC patients, and the other two tsRNA markers (tRF-55:74-chrM.Phe-GAA and tRF-56:75-Ala-CGCC-1-M4) together constitute a new diagnostic signature with the potential to diagnose ESCC (Huang et al., 2023). Certain urinary miRNAs—specifically miR-1273f, miR-619-5p, miR-150-3p, miR-4327, and miR-3135b—exhibited significantly higher levels in patients with EAC. These miRNAs demonstrated high accuracy in distinguishing EAC patients from the HC group, as evidenced by their AUC values approaching 0.80 (Okuda et al., 2021). It has become evident that a variety of miRNAs are potent candidates for diagnostic biomarkers in EC. The selection of miRNA markers is nuanced, with each presenting unique advantages and disadvantages. A critical consideration is the potential of employing a panel of miRNAs or a calculated ratio thereof to enhance diagnostic accuracy. This comprehensive strategy could harness the collective strengths of various miRNAs, compensating for individual weaknesses and offering a robust diagnostic framework.

### 4.2 Prognostic biomarkers in esophageal cancer

In the management of EC, accurate prediction of survival time, disease progression rate, and therapeutic outcomes is crucial for assessing the prognosis of patients. Despite significant advancements in surgical resection, radiotherapy, and chemotherapy for EC, the long-term survival rates remain dismal, with less than 20% of patients surviving beyond 5 years (Sheikh et al., 2023). At present, early detection and secondary prevention of ESCC and EAC subtypes are facing significant challenges. Consequently, miRNAs have become an effective prognostic factor for ESCC patients due to its stable and reliable expression levels in tissues and circulatory systems. For instance, miR-21 expression was significantly higher in patients with poor overall prognosis of ESCC, and miR-21 was expected to serve as an indicator for predicting survival in ESCC patients (Samiei et al., 2022). Furthermore, high expression levels of miR-21 are associated with shorter disease-specific survival (DSS), and this miRNA was identified as an independent prognostic marker for EAC in multivariate analysis (Winther et al., 2015). Meanwhile, miRNAs associated with the prognosis and survival rate of EC patients also include miR-375, miR-25, miR-515-3p, miR-421, miR-146, and miR-550a-1 (Wu K. et al., 2022; Winther et al., 2015; Guo and Tian, 2023; Hu et al., 2020; Ji et al., 2023; Wang et al., 2016; Wang WT. et al., 2019). Among them, high expression of miR-375, miR-515-3p, miR-146 is positively correlated with better prognosis and higher survival rate in patients, and closely related to less metastasis, better staging. Conversely, high expression of miR-25, miR-421, miR-550a-1, and miR-93 is associated with lower survival rates in ESSCC patients. MiR-421 and miR-550a-1 could also serve as indicators of overall survival (OS) in EAC patients. In addition, multiple studies have shown that miR-449b-5p, miR-637, miR-940, miR-4766-5p, miR-455-3p, and miR-214 are closely related to TNM staging and differentiation in ESCC patients (Wang H. et al., 2020; Guo et al., 2022; Wang C. et al., 2022; Zhou PL. et al., 2021; Yang H. et al., 2017; Jia J. et al., 2021). Specifically, low expression of miR-637 was positively correlated with advanced TNM staging, poor differentiation status, and lymph node metastasis (Guo et al., 2022). Wang H. et al. (2020) demonstrated that low miR-940 expression was associated with poor patient prognosis. Cox regression analysis showed that lymph node metastasis, clinical stage, and miR-940 expression were independent risk factors affecting patient prognosis in ESCC. In ESCC patients, low expression of miR-449b-5p was not only associated with better tumor staging and higher differentiation, but also with fewer metastases (Wang C. et al., 2022), while high levels of miR-25 expression leading to a higher likelihood of tumor metastasis (Guo and Tian, 2023). Above-mentioned studies demonstrate that the exploration of new biomarkers related to the prognosis of EC, including differentially expressed miRNAs and their target genes, provides insight into their mechanisms in promoting tumor progression in EC development (Zhao et al., 2021). A comprehensive overview of miRNAs in facilitating diagnosis and prognosis of EC is provided in Table 2.

**TABLE 2:** A comprehensive summary of miRNAs in assisting diagnosis and prognosis of EC.

Source	miRNAs	Diagnosis/Prognosis	Expression	Ref.
Serum	miR-21	Diagnosis/Prognosis	↑	Samiei et al. (2022), Winther et al. (2015)
	miR-375		↓	Winther et al. (2015), Wu et al. (2022b)
	miR-25		↑	Guo and Tian (2023)
	miR-515-3p		↓	Hu et al. (2020)
	miR-940		↓	Wang et al. (2020a)
	miR-421		↑	Ji et al. (2023)
	miR-550a-1		↑	
	miR-196a-5p		↑	Xian et al. (2024)
	miR-205-5p		↑	Kim et al. (2022)
	miR-429		↑	
	miR-375-3p		↓	
	miR-4488	Diagnosis	↑	Huang et al. (2023)
	miR-493-5p		↓	Xiao et al. (2024)
	miR-637	Prognosis	↓	Guo et al. (2022)
Urine	miR-1273f	Diagnosis	↑	Okuda et al. (2021)
	miR-619-5p		↑	
	miR-150-3p		↑	
	miR-4327		↑	
	miR-3135b		↑	

### 4.3 Treatment resistance of esophageal cancer

The rising incidence of EC poses significant challenges to effective cancer therapy, particularly due to the development of treatment resistance. The standard treatment for EC typically involves surgical resection, frequently accompanied by chemotherapy and radiation therapy. Despite substantial progress in anticancer treatments over recent decades, the mechanisms behind multidrug resistance remain not fully understood. In this context, an emerging body of research and preclinical data have highlighted the crucial role of miRNAs in mediating treatment resistance in EC, encompassing both chemotherapy and radiotherapy resistance.

#### 4.3.1 Chemotherapy resistance

Chemotherapeutic agents like 5-fluorouracil (5-FU) and cisplatin (DDP) are frequently utilized, yet drug resistance poses a significant challenge to their therapeutic efficacy and clinical application (Harada et al., 2016). Recent advancements in understanding the mechanisms underlying drug resistance in EC have revealed a correlation between the aberrant expression of miRNAs and chemoresistance. Investigations have identified that miRNAs such as miR-1254, miR-378d, miR-203a, and miR-10a exhibit reduced expression in EC tissues (Takashima et al., 2023; Peng et al., 2021; Wang L. et al., 2020; Hu et al., 2024). These miRNAs modulate the expression of their target mRNAs, influencing EC cell proliferation, migration, invasion, and promoting apoptosis. Overexpression of these miRNAs in EC cells can enhance the therapeutic impact of 5-FU or DDP and improve patient survival rates. Conversely, certain miRNAs, including miR-766-5p, miR-545-3p, and miR-432-3p (Wang L. et al., 2021; Liu et al., 2017; Akdemir et al., 2017), are overexpressed in ESCC, and their antagonists can sensitize ESCC to chemotherapy. Notably, miRNAs primarily contribute to the regulation of DDP and 5-FU resistance by affecting cellular behavior and associated signaling pathways, offering novel strategies and potential molecular targets for EC treatment.

##### 4.3.1.1 Regulating cell proliferation and apoptosis

It has been increasingly recognized that miRNAs are critical regulators of gene expression and play a significant role in modulating cell proliferation and apoptosis in EC. Their ability to target specific genes and pathways can profoundly impact cancer progression and drug responsiveness. For instance, Wang et al. discovered that miR-203a can directly target the key components of the PI3K/AKT/mTOR signaling pathway. By suppressing the expression of these target genes, miR-203a can effectively inhibit the activation of the PI3K/AKT/mTOR signaling pathway, inhibit cell proliferation, thereby affecting the chemical sensitivity of ESCC cells to DDP (Wang L. et al., 2020). Furthermore, MiR-29c directly targets FBXO31 and regulates its expression, inhibiting cell proliferation and enhancing the sensitivity of ESCC to 5-FU (Li et al., 2019). Hu et al. discovered that the overexpression of miR-143 leads to the targeting of CCAT1, which in turn suppresses its expression. This mechanism effectively curbs the proliferation of ESCC cells and enhances their responsiveness to DDP chemotherapy (Hu et al., 2019). MiR-494-3p can directly interact with the circRNA circ_0007142, leading to the degradation of LASP1. This downregulation of LASP1 enhances the sensitivity of DPP-resistant ESCC cells to DPP treatment. Consequently, it promotes apoptosis and invasion while inhibiting cell proliferation (Chang et al., 2022). Moreover, miR-21 promotes DDP resistance in ESCC cells by downregulating PTEN expression (Wan et al., 2021). Shi et al. reported that overexpression of miR-193 in extracellular vesicles can lower adjusting TFAP2C reduces the inhibition of DDP on cyclin in EC cells, promotes cell division and proliferation, and leads to drug resistance (Shi et al., 2020). MiR-106b-3p upregulates in ESCC inhibits cell apoptosis by negatively regulating the expression of TGM3 through binding to 3'UTR sequence of TGM3, inducing ESCC resistance to DDP (Zhu et al., 2021). Liu et al. demonstrated that lncRNA FOXD2-AS1 acts as a competitive endogenous RNA targeting miR-195, promoting ESCC cell growth by activating the AKT/mTOR signaling pathway, inhibiting apoptosis, and developing DDP resistance (Liu et al., 2020b).

##### 4.3.1.2 Affecting cell migration and invasion ability

Wang et al. found that overexpression of miR-455-3p and miR-545-3p promotes the proliferation and migration ability of ESCC cells by inhibiting MT1M expression, and reduces the sensitivity of tumor cells to DDP (Wang L. et al., 2021). Overexpressing of miR-1254 enhances ESCC cells' sensitivity to DPP by downregulating ABCC1and inhibits cell migration and invasion which helps to overcome DPP resistance in ESCC (Takashima et al., 2023). Inhibiting miR-378d has been shown to activate the AKT-β-catenin signaling pathway, which in turn enhances the expression of the EMT marker vimentin and the stem cell marker ALDH1A1. This activation leads to promote the process of EMT and metastasis. Such changes may contribute to the development of a malignant phenotype in ESCC cells and could potentially promote their resistance to chemotherapy (Peng et al., 2021). Yan et al. found that overexpression of miR-624 upregulates YAP expression and activates the HIF1 α signaling pathway by inhibiting ARRDC3 expression, promoting cell migration and invasion ability, and increasing DDP resistance in ESCC cells (Yan et al., 2021). Furthermore, lncRNA NORAD promotes ESCC cell migration and invasion by sponge like adsorption of miR-224-3p, upregulating the expression of MTDH, and leading to DDP resistance (Jia Y. et al., 2021). Exosomes carrying PD-L1 may contribute to paclitaxel resistance by regulating the STAT3/miR-21/PTEN/Akt axis and promoting a tumorigenic phenotype (Wang et al., 2023). In ESCC cells, the expression of miR-194-5p is reduced. Overexpression of miR-194-5p can decrease the protein expression of JMJD1C, inhibit the diffusion and invasion of ESCC cells, thereby enhancing the sensitivity of these cells to paclitaxel (Qu et al., 2021). Chemotherapy is vital for treating EC, but it's challenged by side effects and varying effectiveness. Identifying miRNA biomarkers could pave the way for personalized EC treatments. Future research should aim to combine chemotherapy with other treatments and use biomarkers to tailor protocols, ultimately improving treatment success.

#### 4.3.2 Radiotherapy resistance

Radiotherapy is a crucial modality in the treatment of EC, where it has been instrumental in curbing the proliferation and metastasis of the disease in clinical settings. Nonetheless, the clinical efficacy of radiotherapy has often been hindered by the radioresistance exhibited by cancer cells, which significantly contributes to the suboptimal therapeutic outcomes observed in many EC cases. Recent advancements in miRNA research have shed light on the pivotal role these biomolecules play in the development of radioresistance in EC, offering new strategies to enhance treatment effectiveness.

Several studies have identified specific miRNAs that are downregulated in EC, contributing to radiotherapy resistance. For instance, low expression levels of miR-199a-5p, miR-498, miR-145, miR-485-5p, miR-450a-5p, miR-195-5p, and miR-193b reduce the sensitivity of cancer cells to radiotherapy, leading to poorer treatment outcomes and decreased overall survival rates (Sun et al., 2021; Zhou et al., 2022; Chu et al., 2024; Wang M. et al., 2020; Wang J. et al., 2021; Chen H. et al., 2020; Chai et al., 2024; Jin et al., 2020). MiR-199a-5p can suppress the ATR/Chk1 signaling pathway by directly targeting EEPD1, thereby restoring radiosensitivity in radiotherapy-resistant cancer cells (Sun et al., 2021). Similarly, Zhou et al. found that miR-498 enhances the sensitivity of EC cells to radiation by inhibiting DNMT3b and inactivating the PI3K/AKT signaling pathway (Zhou et al., 2022). MiR-876-5p directly targets the proto-oncogene tyrosine-protein kinase (FYN), and its upregulation significantly inhibits FYN protein expression, reducing radiotherapy tolerance (Chu et al., 2024). In addition, miR-145 targets the p70S6K1 protein, and its overexpression diminishes p70S6K1 levels, suggesting that miR-145 may augment radiosensitivity by suppressing this protein (Wang M. et al., 2020). Additionally, miR-145 is involved in the p53 signaling pathway, with increased p53 protein expression linked to higher miR-145 levels, further enhancing radiosensitivity (Wang M. et al., 2020). Wang et al. demonstrated that miR-485-5p enhances radiosensitivity by suppressing FERMT1 expression and modulating the FERMT1 signaling pathway. This discovery implies that miR-485-5p could be a potential therapeutic target for improving the effectiveness of radiotherapy (Wang J. et al., 2021). MiR-450a-5p amplifies the p38 and SAPK/JNK signaling pathways by suppressing DUSP10, a phosphatase that regulates apoptosis, thereby counteracting radiotherapy resistance (Chen H. et al., 2020). Chai et al. identified that miR-195-5p targets SPIN1, and by suppressing its expression, it diminishes radiotherapy resistance in ESCC (Chai et al., 2024). Moreover, miR-193b targets the 3'UTR of Cyclin D1 mRNA, inhibiting its expression and reversing the cell cycle arrest at the G0/G1 phase in radiotherapy-resistant EC cells, thus enhancing radiosensitivity (Jin et al., 2020).

Conversely, some miRNAs promote radioresistance in EC. For example, miR-4443 enhances radioresistance in ESCC by directly targeting PTPRJ and upregulating the targeted protein (Shi et al., 2022). Gao et al. found that tumor-derived extracellular vesicle miR-143-3p induces M2 polarization in macrophages, promoting radiation resistance in locally advanced ESCC (Gao et al., 2024). Similarly, miR-494 also enhances radiation resistance by inhibiting PD-L1 degradation (Li et al., 2024). It can be seen that multiple miRNAs affect the sensitivity of EC cells to chemotherapy and radiotherapy. These miRNAs can serve as targets for future clinical treatment and are expected to assist in the treatment of EC, improving the survival and quality of life of EC patients. The involvement of mRNA in the regulation of chemotherapy and radiotherapy resistance in EC and its mechanisms are detailed in Table 3.

**TABLE 3:** The relationship between miRNAs and chemotherapy/radiotherapy resistance in EC.

Promoting/inhibiting treatment resistance	miRNAs	Expression	Target	Ref.
Inhibiting chemotherapy resistance	miR-203	↓	PI3K/AKT/mTOR↓	Wang et al. (2020b)
	miR-29c		FBXO31↓	Li et al. (2019)
	miR-143		CCAT1↓	Hu et al. (2019)
	miR-494-3p		LASP1↓	Chang et al. (2022)
	miR-378d		AKT-β-catenin↑	Peng et al. (2021)
	miR-224-3p		MTDH↓	Jia et al. (2021b)
	miR-194-5p		JMJD1C↓	Qu et al. (2021)
Promoting chemotherapy resistance	miR-195	↑	AKT/mTOR↑	Liu et al. (2020b)
	miR-455-3p		MT1M↓	Liu et al. (2017)
	miR-545-3p			Wang et al. (2021b)
	miR-1254		ABCC1↓	Takashima et al. (2023)
	miR-21		PTEN↓	Wan et al. (2021)
	miR-624		ARRDC3↓	Yan et al. (2021)
	miR-193		TFAP2C↓	Shi et al. (2020)
	miR-21		STAT3↑	Wang et al. (2023)
	miR-106b-3p		TGM3↓	Zhu et al. (2021)
Inhibiting radiotherapy resistance	miR-199a-5p	↓	EEPD1↓	Sun et al. (2021)
	miR-498		DNMT3b↓	Zhou et al. (2022)
	miR-876-5p		FYN↓	Chu et al. (2024)
	miR-145		p70S6K1↓ p53↑	Wang et al. (2020c)
	miR-485-5p		FERMT1↓	Wang et al. (2021c)
	miR-450a-5p		DUSP10↓	Chen et al. (2020c)
	miR-195-5p		SPIN1↓	Chai et al. (2024)
	miR-193b		Cyclin D1↓	Jin et al. (2020)
Promoting radiotherapy resistance	miR-4443	↑	PTPRJ↓	Shi et al. (2022)
	miR-494		PD-L1↑	Li et al. (2024)

### 4.4 Novel treatment of esophageal cancer

#### 4.4.1 Targeted therapy

Due to the intricate pathogenic mechanisms associated with EC, the majority of patients receive a diagnosis at intermediate to advanced stages, precluding the possibility of timely radical surgical intervention. Conventional radiotherapy and chemotherapy, while effective in eradicating tumor cells, also inflict substantial harm on normal cells, leading to severe toxic side effects (Mao et al., 2021). The advent of molecular targeted therapy has addressed several limitations inherent in traditional treatment modalities for EC, thereby offering patients a broader range of therapeutic options. Molecular targeted therapy involves the use of pharmacological agents that specifically target molecules at the cellular and molecular levels, thereby inhibiting the proliferation and dissemination of cancer cells. These agents, often referred to as “biological missiles,” bind precisely to identified oncogenic targets upon administration (Fu et al., 2022). The identification of an optimal target is essential for the efficacy of molecular targeted therapy in cancer treatment. Typically, genes that differentiate cancerous cells from normal cells are considered suitable targets for the development of molecular targeted drugs. Presently, miRNAs have shown potential in treating EC by targeting molecular entities such as CXCL10, PDCD4, TIMP3, TCTN1, and CCND1.

Recent studies have elucidated the involvement of miRNAs in EC, providing novel insights into potential therapeutic strategies. Zhang et al. identified that miR-21-5p is transmitted via exosomes in ESCC, where it targets the inhibition of CXCL10. This interaction facilitates angiogenesis and accelerates tumor progression, implying that the inhibition of miR-21-5p or the disruption of its interaction with CXCL10 could be effective in suppressing tumor growth and angiogenesis (Zhang et al., 2022b). Furthermore, miR-21 is upregulated in various cancers, including EC, where it targets tumor suppressor genes such as PDCD4 and TIMP3, resulting in enhanced tumor cell proliferation and survival. Thus, inhibiting miR-21 or obstructing its interaction with these genes could potentially mitigate tumor development (Kan and Meltzer, 2009). Chai et al. identified miR-216a-5p as a tumor suppressor in ESCC cells by negatively regulating TCTN1 expression, indicating that miR-216a-5p and TCTN1 may serve as promising targets for ESCC therapeutic interventions (Chai and Yang, 2019). Furthermore, Jiang et al. demonstrated that miR-503 predominantly functions in tumor suppression by targeting the CCND1 gene in ESCC, thereby regulating cell cycle progression and inhibiting cell proliferation, migration, and invasion. This finding suggests that miR-503 could be a viable target for therapeutic strategies aimed at controlling tumor growth and metastasis (Jiang et al., 2017). These studies highlight the potential of miRNAs as therapeutic targets in EC. By understanding and manipulating these miRNA pathways, researchers can develop more effective and targeted treatments, improving outcomes for patients with this challenging form of cancer.

#### 4.4.2 Immunotherapy

In addition to focusing on intrinsic signaling pathways within EC cells, immunotherapy has emerged as a significant area of research in the treatment of EC. This therapeutic approach primarily aims to enhance the functionality and specificity of immune cells to inhibit cancer progression. Typically, antigen-presenting cells, particularly dendritic cells, recognize and process tumor-associated antigens on the surface of cancer cells, subsequently presenting them to T or B lymphocytes to initiate an adaptive immune response. This adaptive immune response is crucial for the elimination of tumor cells. However, tumor cells can evade immune detection and destruction through various mechanisms. For instance, they may express immune checkpoint proteins that suppress T cell activity, thereby escaping T cell-mediated surveillance and attack (Park et al., 2024). In recent years, the development of immunotherapy for EC has advanced rapidly, with the goal of restoring the recognition and cytotoxic capabilities of immune cells to counteract tumor cell immune evasion, thereby allowing the immune response to proceed effectively. Currently, immunotherapeutic strategies for EC primarily target the PD-1, PD-L1, and CTLA-4 pathways. MiRNAs play a crucial role in EC immunotherapy by modulating these immune checkpoints, influencing the tumor microenvironment, and serving as key regulators.

Recent research has underscored the critical role of miRNAs in modulating immune responses in EC. Zhang et al. elucidated that miR-145-5p targets and suppresses the expression of SPOP, which subsequently facilitates the ubiquitination and degradation of PD-L1. This process results in elevated PD-L1 levels, enabling its interaction with PD-1 and the subsequent inhibition of T cell activity, thereby promoting immune evasion by the tumor (Zhang et al., 2021). Inhibition of miR-145-5p may, therefore, enhance T cell cytotoxicity against EC cells. Sudo et al. (2020) demonstrated that miRNAs have the potential to predict the therapeutic response of EC patients to nivolumab, indicating that miRNAs may influence the interaction between immune cells and tumor cells by modulating immune checkpoint molecules such as PD-1 and CTLA-4. For instance, certain miRNAs can downregulate PD-1 expression, thereby augmenting T cell-mediated anti-tumor activity. Wang et al. reported that miR-21 targets and inhibits the PTEN gene, thereby activating the Akt signaling pathway, which promotes tumor cell survival, proliferation, and immune evasion. Furthermore, PD-L1 enhances miR-21 expression by increasing STAT3 binding to the miR-21 promoter (Wang et al., 2023). Li et al. discovered that miR-494-3p can inhibit the degradation of PD-1, thereby attenuating the immune response (Li et al., 2024). Pengjie et al. discovered that miR-378a-5p participates in ceRNA network regulation by targeting key genes APOC1 and CEP55, indirectly affecting immune cell infiltration and function, and exerting tumor suppressive effects in immunotherapy of ESCC (Pengjie et al., 2024). These findings underscore the potential of miRNAs as therapeutic targets in enhancing the immune response against EC. By modulating the expression of specific miRNAs, it may be possible to overcome immune evasion mechanisms and improve the efficacy of immunotherapy in EC.

## 5 Conclusion and perspectives

In this review, we have comprehensively examined the complex role of miRNAs in EC, highlighting their potential as novel biomarkers for diagnosis, prognosis, and treatment resistance. We underscore the profound impact of miRNAs on the pathogenesis of EC, particularly their influence on cellular processes such as proliferation, apoptosis, angiogenesis, and metastasis. The review reveals that miRNAs are integral to the pathogenesis of EC and may serve as viable targets for therapeutic intervention. The distinct expression patterns of miRNAs in EC have been instrumental in identifying potential diagnostic and prognostic markers, which hold the potential to transform patient management through early intervention and personalized treatment strategies.

As we reflect on the critical role of miRNAs in EC and their promise as biomarkers, it becomes evident that further exploration is essential to fully harness their potential in clinical settings. The following research directions should be considered: (1) The interaction between miRNAs and other non-coding RNAs, such as lncRNAs and circRNAs, adds another layer of complexity to the regulatory networks in EC. Elucidating these interactions will provide insights into the fine-tuning of gene expression and may reveal novel therapeutic avenues. (2) The role of miRNAs within the tumor microenvironment, including their influence on immune cells and angiogenesis, warrants further exploration, as it may offer new strategies for immune checkpoint inhibitors or combinatorial therapies. Investigate the interplay between miRNAs and immune checkpoints in EC, potentially uncovering new biomarkers for immunotherapy response. (3) Single-cell sequencing can provide insights into the immune microenvironment. Future research should leverage this technology to uncover novel miRNA-associated pathways in EC. (4) Bridge the gap between miRNA discoveries and clinical applications, exploring the potential of miRNAs in liquid biopsies for real-time monitoring of EC progression and treatment response. (5) Use organoid cultures to study the role of miRNAs in EC clonality and treatment response, which could inform precision medicine approaches. (6) The association of genetic variations in the miRNA regulatory pathway with EC risk or outcome is a largely uncharted territory. Future genetic epidemiological studies should explore these variations to understand their role in EC development and prognosis.

In conclusion, the intersection of miRNA research with the latest scientific advancements offers a rich landscape for future exploration in EC. It is imperative that our research efforts are directed towards these emerging areas to translate theoretical insights into tangible clinical benefits, ultimately improving patient outcomes and quality of life for those affected by EC.
